# An Alignment-Free Approach for Eukaryotic ITS2 Annotation and Phylogenetic Inference

**DOI:** 10.1371/journal.pone.0026638

**Published:** 2011-10-26

**Authors:** Guillermin Agüero-Chapin, Aminael Sánchez-Rodríguez, Pedro I. Hidalgo-Yanes, Yunierkis Pérez-Castillo, Reinaldo Molina-Ruiz, Kathleen Marchal, Vítor Vasconcelos, Agostinho Antunes

**Affiliations:** 1 CIMAR/CIIMAR, Centro Interdisciplinar de Investigação Marinha e Ambiental, Universidade do Porto, Porto, Portugal; 2 Molecular Simulation and Drug Design (CBQ), Universidad Central “Marta Abreu” de Las Villas (UCLV), Santa Clara, Cuba; 3 Departamento de Biologia, Faculdade de Ciências, Universidade do Porto, Porto, Portugal; 4 CMPG, Department of Microbial and Molecular Systems, KU Leuven, Leuven, Belgium; 5 Area of Microbiology, University of León, León, Spain; J. Craig Venter Institute, United States of America

## Abstract

The ITS2 gene class shows a high sequence divergence among its members that have complicated its annotation and its use for reconstructing phylogenies at a higher taxonomical level (beyond species and genus). Several alignment strategies have been implemented to improve the ITS2 annotation quality and its use for phylogenetic inferences. Although, alignment based methods have been exploited to the top of its complexity to tackle both issues, no alignment-free approaches have been able to successfully address both topics. By contrast, the use of simple alignment-free classifiers, like the topological indices (TIs) containing information about the sequence and structure of ITS2, may reveal to be a useful approach for the gene prediction and for assessing the phylogenetic relationships of the ITS2 class in eukaryotes. Thus, we used the **TI2BioP** (**T**opological **I**ndices **to BioP**olymers) methodology [1], [2], freely available at http://ti2biop.sourceforge.net/ to calculate two different TIs. One class was derived from the ITS2 artificial 2D structures generated from DNA strings and the other from the secondary structure inferred from RNA folding algorithms. Two alignment-free models based on Artificial Neural Networks were developed for the ITS2 class prediction using the two classes of TIs referred above. Both models showed similar performances on the training and the test sets reaching values above 95% in the overall classification. Due to the importance of the ITS2 region for fungi identification, a novel ITS2 genomic sequence was isolated from *Petrakia* sp. This sequence and the test set were used to comparatively evaluate the conventional classification models based on multiple sequence alignments like Hidden Markov based approaches, revealing the success of our models to identify novel ITS2 members. The isolated sequence was assessed using traditional and alignment-free based techniques applied to phylogenetic inference to complement the taxonomy of the *Petrakia* sp. fungal isolate.

## Introduction

Standard alignment methods are less effective for the functional prediction of gene and protein classes that show a high primary sequence divergence between their members [3]. Thus, the implementation of stochastic models [4], the modification of the original similarity matrixes among the aligned sequences, and the addition of other steps in the alignment procedures [5], [6], have been strategies adopted to improve the classification of divergent gene/protein functional classes. On the other hand, several alignment-free methods have been developed as an alternative to traditional alignment algorithms for gene/protein classification at low sequence similarity level [1], [7], [8].

The internal transcribed spacer 2 (ITS2) eukaryotic gene class is one of the cases showing a higher sequence divergence among its members, which have traditionally complicated ITS2 annotation and limited its use for phylogenetic inference at low taxonomical level analyses (genus and species level classifications). Despite the ITS2 high sequence variability, the ITS2 structure has been considerably conserved among all eukaryotes [9]. This fact has been considered for the implementation of homology-based structure modelling approaches to improve the ITS2 annotation quality and also as a tool for eukaryote phylogenetic analyses at higher classification levels or taxonomic ranks [6], [9], [10]. Thus, the ITS2 database (http://its2.bioapps.biozentrum.uni-wuerzburg.de) was developed holding information about sequence, structure and taxonomic classification of all ITS2 in GenBank [11]. However, due to ITS2 high sequence variability, the annotation pipeline implemented in the aforementioned resource requires the use of a specific score matrix in the BLAST search [11] and more recently, the use of HMM for the identification and delineation of the ITS2 sequences [10], [12]. Although alignment based methods have been exploited to the top of its complexity to tackle the ITS2 annotation and phylogenetic inference [10], [11], no alignment-free approach has been able to successfully address these issues so far. The use of simple alignment-free classifiers like the topological indices (TIs) containing also information about the sequence and structure of ITS2 can be another useful approach for the prediction and phylogenetic analyses of the ITS2 class in eukaryotes. Such TIs are determined by our methodology entitled **T**opological **I**ndices **to BioP**olymers **“TI2BioP”** where the spectral moments are calculated from different graphical approaches representing the structure of the biopolymers: DNA, RNA and proteins [1], [2]. TI2BioP is now available at http://ti2biop.sourceforge.net/ as a public tool for the calculation of two different TIs, one class derived from the ITS2 artificial 2D structures generated from DNA strings (Nandy structures) [13], [14] and the other class resulting from the secondary structure inferred with RNA folding algorithms (Mfold) [15]. These alignment-free classifiers were used to build linear and Artificial Neural Networks (ANN)-models for classifying the ITS2 members among positive and negative sets and also to estimate the ITS2 phylogeny at higher classification levels.

The ANN-models provided the highest classification accuracy (95.9 and 97.5%) during the training step compared to the linear models for Nandy-like and Mfold structures, respectively. A very similar ANN performance was obtained for the test set for both structural representations. These results support that the identification of gene signatures tend to be better when assessed with non-linear models. We also showed the utility of the artificial secondary structure when the correct 2D structure is not available (i.e. the physiological structure that occurs on the cell) and can only be obtained by predictions based on free energy minimizations.

The performance of our two alignment-free models based on ANN was also compared with several profile Hidden Markov Models (HMMs) generated from alignments performed with CLUSTALW [16], DIALIGN-TX [17] and MAFFT [18] using different training sets, to classify the test set and to identify a new fungal member of the ITS2 class. Moreover, a BLASTn search against NCBI was carried out to give more reliability to the gene annotation and to assess taxonomically related hits to our query fungal sequence. ITS2 is the standard gene target for fungal identification and taxonomy at the species level [19]. This new ITS2 sequence was isolated by our group (GenBank accession number FJ892749) from an endophytic fungus belonging to the genus *Petrakia*. Members of this fungal genus have been hard to be placed taxonomically and are potential producers of bioactive compounds [20]. The *Petrakia sp.* strain was morphologically identified and its ITS2 sequence was used to carry out traditional and alignment-free phylogenetic analyses to support its taxonomic characterization.

The alignment-free models identified the new query sequence as a member of the ITS2 class with high significance, while the profile HMMs showed a poor performance in doing so. We demonstrated that our TIs are useful not only in sequence identification but also in molecular evolutionary inferences. The alignment-free tree built based on TIs provided similar phylogenetic relationships among the different classes of the Ascomycota phylum in respect to the traditional phylogenetic analysis (i.e. based on evolutionary distances derived from a multiple alignment of DNA sequences). Both analyses placed the *Petrakia* genus inside the *Pezizomycotina* subphylum and the *Dothideomycetes* class.

## Methods

### 1. Computational methods. Topological Indices to BioPolymers (TI2BioP)

TI2BioP allows the calculation of the spectral moments derived from inferred and artificial 2D structures of DNA, RNA and proteins [21]. Consequently, it is feasible to carry out a structure-function correlation using such sequence/structure numerical indices. The calculation of the spectral moments as sequence descriptors is performed according to the TOPS-MODE approach [22] implemented in the “MODESLAB” software [23] and the draw mode for sequence representation was retrieved from the MARCH-INSIDE methodology [24], [25], [26]. TI2BioP can also import files containing 2D structure inferred by other professional softwares like the RNASTRUCTURE [15]. We propose for the first time to fold the ITS2 genomic sequences into an artificial secondary structure based on Nandy's representation for DNA strings [13]. This graph groups purine and pyrimidine bases on a Cartesian system assigning to X and Y axes each nucleotide-type, respectively. The representation was carried out by adding to the coordinates (0, 0) of the Cartesian system the k-th nucleotide of the DNA sequence. The value (1, 0) if the (k+1)-th nucleotide is Guanine (rightwards-step); (−1, 0) if Adenine (leftwards-step); (0, 1) if Cytosine (upwards-step) or (0, −1) if the (k+1)-th nucleotide is Thymine or Uracil (downwards-step).

**Figure 1:**
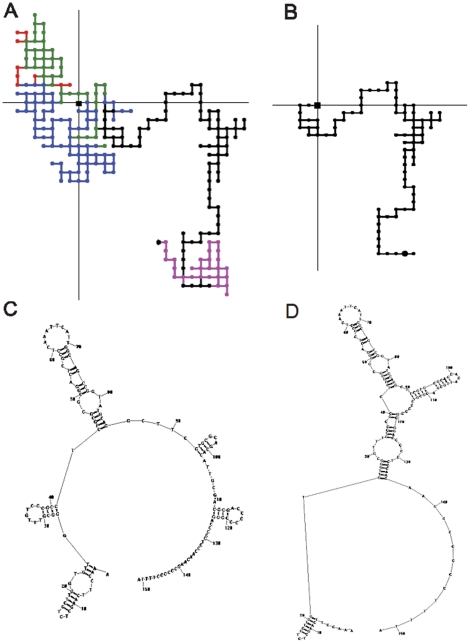
The ITS2 region (in black) with its boundaries ordered 5′upstream: a short end corresponding to the 18S rDNA (in red), the ITS1 (in green), the 5.8S rDNA and 3′downstream: a short fragment of the 28S rDNA (in pink) (A). The ITS2 region pseudo-folded into the 2D-Cartesian system (B). The ITS2 sequence folded as a DNA and RNA structure by the Mfold program, respectively (C and D).

**Figure 1** depicts the 2D Cartesian representation of the 558 bp genomic DNA fragment from *Petrakia sp.* ef08-038 (accession number FJ892749) comprising the ITS2 with its boundaries (**fig. 1A**) and only the ITS2 (**fig. 1B**). The figure also shows the ITS2 sequence (without its boundaries) folded as DNA (**fig. 1C**) and RNA (**fig. 1D**) by the Mfold program.

In the study, a total of 4,355 out of the original 5,092 ITS2 sequences from a wide variety of eukaryotic taxa (http://its2.bioapps.biozentrum.uni-wuerzburg.de) shared similar secondary structure features and were used as positive dataset.

The negative set or control group comprises diverse but structurally related genomic sequences to the ITS2 class: the untranslated regions (UTRs) of eukaryotic mRNAs. They are non-coding regions with divergence among the eukaryotes but showing a more conserved secondary structure when are transcribed into RNAs [27]. A non-redundant subset containing 6,529 and 8,128 of the 5′- and 3′-UTRs' sequences from the fungi kingdom, respectively, was selected from the eukaryotic mRNAs database: UTRdb (http://www.ba.itb.cnr.it/UTR/). The sequence diversity among the ITS2 and UTRs datasets was explored comparatively using the Needleman-Wunsch (NW) [28] and Smith-Waterman (SW) [29] algorithms. See in supporting information (S) the NW & SW analyses (**File S1 and figure S1**).

Training and test series were randomly selected. The members of the test set were selected taking out at random the 20% of the overall data (19,012 cases). The remainder of the cases was used to train the model. Sequences with ambiguity at least in one nucleotide position were removed from both groups. Each ITS2 and UTR sequence retrieved was labeled respecting its original database ID code; see File S2.

All sequences (positive and negative sets) were pseudo-folded into a Cartesian system by TI2BioP to obtain the artificial secondary structures as it was described above. On the other hand, they were also used to infer optimized DNA secondary structures by the Mfold algorithm implemented in the RNASTRUCTURE 4.0 software [30] (**fig. 1C**). The structural optimization is based on the minimization of the folding energy (lowest ΔG). Spectral moments (μ_k_) introduced previously by Estrada et al. (1996) [31], [32] were applied to codify the new structural information contained into the artificial secondary structures and into the inferred secondary structures of both the ITS2 and UTRs sequences.

#### 1.1. Calculation of TIs irrespective of sequence similarity

The topological indices called “spectral moments” were calculated as the sum of the entries placed in the main diagonal of the bond adjacency matrix (**B**) for the DNA/RNA sequences. **B** is a square matrix of n×n row and column where its non-diagonal entries are ones or zeroes if the corresponding bonds or edges share or not one nucleotide. Thus, it set up connectivity relationships between the nucleotides in certain DNA/RNA graph. The different powers of **B** give the spectral moments of higher order.

In the DNA/RNA artificial secondary structure, the number of edges (e) in the graph is equal to the number of rows and columns in **B** but may be equal or even smaller than the number of bonds in the nucleotide sequence. The main diagonal entries of **B** were weighted with the average of the electrostatic charge (Q) between two bound nodes. The charge value q in a node is equal to the sum of the charges of all nucleotide placed on it. The electrostatic charge of one nucleotide was derived from the Amber 95 force field [33]. Thus, it is easy to carry out the calculation of the spectral moments of **B** in order to numerically characterize the pseudo-folding (^pf^ **μ**_k_) of DNA/RNA sequences.
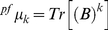
(1)Where Tr is called the trace and indicates the sum of all the values in the main diagonal of the matrices ^k^ **B** = (**B**)^k^, which are the natural powers of **B**.

**Figure 2:**
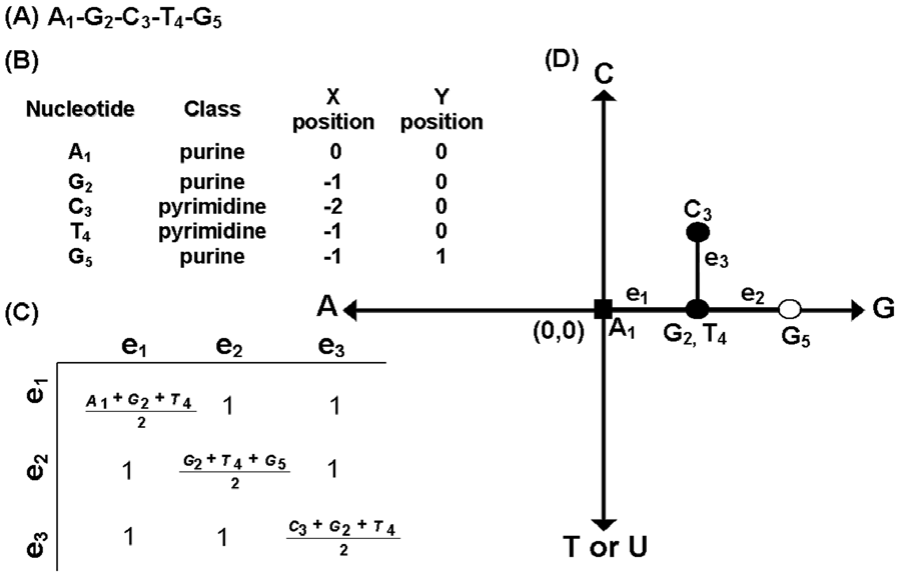
Building the 2D-Cartesian map for the (A) DNA fragment AGCTG. (B) The coordinates for each nucleotide in the Cartesian system. (C) The definition of the bond adjacency matrix derived from (D) the 2D-Cartesian map. Note that all edges of the graph are adjacent, thus all non-diagonal entries are ones.

In order to illustrate the calculation of the spectral moments, an example is developed below. The 2D Cartesian network of the sequence (AGCTG) is showed in the figure 2D and its bond adjacency matrix is depicted in the figure 2C; note that the central node contains both Guanine and Thymine nucleotides. The calculation of the spectral moments up to the order k = 3 is also defined below on the **figure 2**. The q values are represented in the matrix as the nucleotides symbols (G = 0.24, A = 0.22, C = 0.19, T and U = 0.21).

Expansion of expression (1) for k = 1 gives the ^pf^μ_1_, for k = 2 the ^pf^μ_2_ and for k = 3 the ^pf^μ_3_. The calculation of the spectral moments up to order three from this DNA graph is described below.
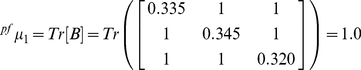
(1a)

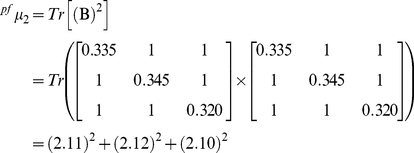
(1b)

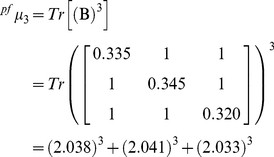
(1c)


**TI2BioP** *version 1.0* ® arrange automatically the DNA/RNA sequences into a 2D Cartesian network [21] and also import the connectivity table (ct files) generated by the RNASTRUCTURE 4.0 software. Ct files contain information about the connection between nucleotides in the secondary structure generated with thermodynamic models [30]. Thus, it is possible to perform the calculation of the spectral moments (^mf^ **μ**_k_) based on folding thermodynamics parameters for the positive and negative sets. Another two additional TIs defined as Edge Numbers and Edge Connectivity were introduced for these two DNA/RNA structural approaches; see File S2 for more details.

### 2. Building up alignment free-models with TIs

#### 2.1. Variable screening

We used the *Feature Selection and Variable Screening* module of the *Data Mining* menu from *STATISTICA* software [34] to select a subset of predictors that is most strongly related to the dependent (outcome) variable of interest regardless of whether that relationship is simple (linear) or complex (nonlinear). The algorithm for selecting those variables is not biased in favor of a single method for subsequent analyses; further post-processing algorithms were applied, based on linear and non-linear modeling methods.

#### 2.2. Alignment-free models for ITS2 classification. Linear models

The General Discrimination Analysis (GDA) was carried out for building up linear models for ITS2 alignment-free identification [35], [36], [37], [38]. The most significant predictors obtained from the variable screening method for each structural approach were used to fit linear discriminant functions. Both subsets of TIs were standardized in order to become equally scaled to allow an effective comparison between the regression coefficients [39]. The model performance was evaluated by several statistical measures: accuracy, area under the Receiver Operating Characteristic (ROC) curve, commonly known as AUC with a value of 1.0 for a perfect predictor and 0.5 for a random predictor and the F-score (it reaches its best value at 1 and worst score at 0) [40].

#### 2.3. Alignment-free models for ITS2 classification. Non-linear models. Artificial Neural Networks (ANN)

We used ANN method for ITS2 classification using the same series of TIs as input variables and only one output variable (ITS2 membership). We used the Multilayer Layer Perceptron (MLP) due to its ability to model functions of almost arbitrary complexity showing a simple interpretation as a form of input-output model. To select the right complexity of the network, we tested different topologies to the MLP while checking the progress against a selection set to avoid over-fitting during the two-phase (back propagation/conjugate gradient descent) training algorithm [41]. The selection set was extracted at random from the training set (10%) by also generating random numbers. The test set was the same used for GDA representing an external subset (not used during training algorithms) to check the final network performance.

The optimal cutoff for ITS2 gene classification for ANN-models was defined by determining on the ROC-curve the model's parameter values (‘accept’ and ‘reject’ classification thresholds) giving the nearest point (optimal operating point) to the (0,1) coordinates. This point constitutes the ideal condition for ITS2 classification (most balanced solution where both specificity and sensitivity are maximized). The optimal operating point was determined by computing the slope S that considers the misclassification costs for each class. The point was found by moving the straight line with slope S from the upper left corner of the ROC plot (0, 1) down and to the right until it intersects the ROC curve.

### 3. Alignment-based models for ITS2 classification. Profile Hidden Markov Models (HMM)

Three training subsets were selected to build up several profile HMMs for ITS2 gene classification: (i) 134 sequences extracted representatively from the original training set (2802 ITS2 sequences) to represent evenly the whole range of sequence similarity while retaining representative members from all the eukaryotic taxa within the training set (this sampling was based on the sequence similarity clustering carried out in File S1); (ii) 80 sequences representative of the fungal kingdom selected following a similar procedure as described in (i); and (iii) 2802 ITS2 sequences used to train the alignment-free models. In addition, three different multiple sequence alignments (MSA) algorithms were used to align these subsets: CLUSTALW [16], DIALIGN-TX [17] and MAFFT [18]. Due to the low similarity level amongst the ITS2 sequences, we have used DALIGN-TX and MAFFT that are expected to outperform CLUSTALW in such conditions. DALIGN-TX is a segment-based multiple alignment tool improved for sets of low overall sequence similarity and the MAFFT program is able to identify homologous regions among distantly related sequences. Performing a good alignment is a crucial step to generate a profile HMM with high classification power.

CLUSTALW and DIALIGN-TX were run using the default parameters. In the case of MAFFT the iterative alignment option (L-INS-I) was used [29], [42].

Alignments were edited in every case as follows: aligned positions were removed from both ends until gaps were observed in less than 10% of the aligned sequences. Thus, we removed non-informative positions from the multiple alignments that could deteriorate the resulting HMM. Edited alignments were used as input for *hmmbuild* release 2.3.2 [43], which generated the profile HMMs. During the profile HMMs generation step the *fast* option of the *hmmbuild* program was used with a default value equal to 0.5. This option assigns the *insert* state to every column in the alignment containing gaps in at least half of the sequences. In this way, the resulting HMMs do not make an explicit use of the sequence distribution (i.e. nucleotides frequencies) of positions with high amount of gaps but rather consider them as insertion states.

The obtained profile HMMs allowed to classify members of the test set, as well as the newly isolated ITS2 sequence from *Petrakia* sp. (see below) using *hmmsearch*. An optimal cutoff for the ITS2 classification was determined by running each profile HMM at 20 different E-values (0.1–10). The E-value that maximizes both sensitivity and specificity was selected as the optimal classification cutoff. The performance of these models at the optimal classification cutoff was compared to that of the alignment-free models described above (sections 2.2.2 and 2.2.3).

### 4. Phylogenetic analyses

We defined an empirical threshold of ITS2 representatives with more than 60% of sequence similarity with our query fungus (Petrakia sp. ef08-038) among the members of the Ascomycota phylum for the phylogenetic analysis. This allowed the retrieval of an ITS2 subset comprising 16 sequences that encompassed several classes from the subphyla Pezizomycotina (Dothideomycetes, Lecanoromycetes, Leotiomycetes and Sordariomycetes), while the remaining cases were either taxonomically characterized as mitosporic Ascomycotas (asexual species that produce conidia namely mitospores) or unclassified Ascomycotas. The 16 ITS2 sequences plus our query sequence (FJ892749) were aligned with the CLUSTAL W setting a Gap Open Penalty (GOP) of 20 and a Gap Extension Penalty (GEP) of 10. The final alignment was edited removing end gaps and the phylogenetic analyses were conducted in MEGA4 software [19]. Neighbour-joining (NJ) trees were generated from different sequence distance matrices from (1) alignment and (2) alignment-free approaches:

1. NJ trees based on different evolutionary distances computed using Jukes-Cantor (JC), Kimura 2-parameter (K2P) and Maximum Composite Likelihood (MCL) substitution models were obtained using the MEGA4. In addition, the Minimum Evolution (ME) method was assessed on the JC and K2P distance matrices. The bootstrap support (BS) values for nodes were computed from 1000 replicates.
2. A NJ tree was built based on the hierarchic clustering that uses the Euclidean distance matrix as a multidimensional measure to form the sequences clusters. Euclidean distance (Ed) was computed from the TIs values of the same seventeen ITS2 sequences mentioned above and the complete linkage or furthest neighbor was used as cluster method.
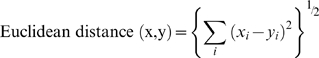
(2)The quality of this numerical taxonomy was tested (i) performing the Joining Tree Clustering with different distance metrics (City-block, Chebychev, and Power distance), (ii) using other cluster methods (Single linkage, Unweighted pair-group average and the Ward's method), and (iii) calculating the cophenetic correlation coefficient.

### 5. Experimental section

*Petrakia* strain was isolated from leaves of *Acer psedoplatanus*. The plant material was collected in Kaiserslautern, Germany. It was cut and surface-sterilized by immersion in 70% ethanol for 1 min, 5% NaOCl for 3 min and 70% ethanol for 1 sec followed by a wash in sterile distilled water. Samples were then cut into small fragments and plated onto 2% malt agar with penicillin G and streptomycin sulfate (each 200 mg/l). The mycelial culture was deposited in the culture collection of the Institute of Biotechnology and Drug Research (IBWF), Kaiserslautern.

DNA extraction was performed as described previously by Sacks [44]. The entire ITS (ITS1, 5.8S rDNA, and ITS2) region was amplified for ITS sequence analysis. The primers used for amplification were ITS5 (5′-GGAAGTAAAAGTCGTAACAAGG) and ITS4 (5′- TCCTCCGCTTATTGATATGC) according to White et al. [45]. Their method was used with slight modifications: A GeneAmp PCR System 9700 was employed (Applied Biosystem, Foster City, CA, USA). The PCR amplification cycle consisted of 30 s at 94°C, 1 min at 50°C, and 1 min at 72°C. PCR products were sequenced by MWG Biotech (Ebersberg, Germany) with the same primers used for the amplification. Each sequence was obtained in duplicate from each of two separate PCR amplifications.

## Results and Discussion

### 6. Predicting eukaryotic ITS2 sequences with alignment-free classifiers

Two classes of predictors comprising 18 TIs each were calculated by the TI2BioP methodology for 19,012 genomic sequences (4,355 ITS2 and 14,657 UTRs): the spectral moments series (**μ**_0_- **μ**_15_) of the bond adjacency matrix between the nucleotides arranged into the Cartesian space (^pf^ **μ**_k_) and between the nucleotides connected into the Mfold structures (^mf^ **μ**_k_). Other two additional TIs were computed (the Edge Numbers and the Edge Connectivity) for each class. The spectral moments are structural-based TIs that describe electronically the nucleotide connectivity at different orders in these two structural approaches. The Nandy-like structure is determined by the sequence order and DNA/RNA nucleotide composition. The 2D structure obtained by the Mfold software depends also of the primary sequence but its folding is driven by the optimization of thermodynamics parameters (lowest folding free energy-ΔG^0^).

**Figure 3:**
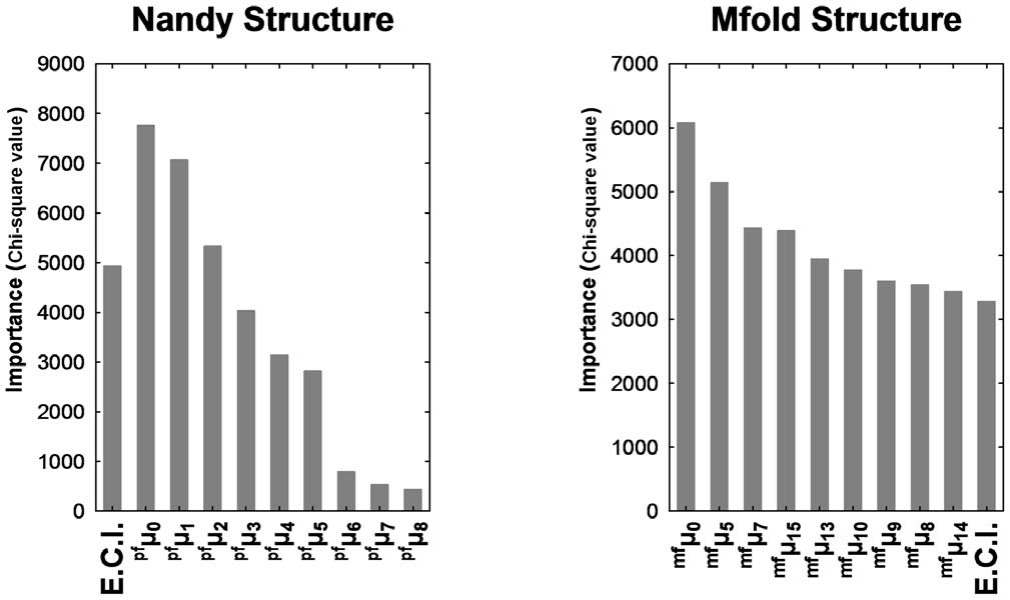
Predictor importance according the variable screening analysis for the Nandy and Mfold structures. E.C.I. (Edege Connectivity Index).

In order to select the most significant predictors for both datasets (Nandy-like and Mfold structures), we carried out a feature selection as a preliminary variable screening method before the model building. We found that the four most significant variables (p<0.01) were the Edge Connectivity, the ^pf^μ_0_, ^pf^μ_1_, and ^pf^μ_2_ for Nandy's structures and for Mfold structures the ^mf^μ_0_, ^mf^μ_5_, ^mf^μ_7_ and ^mf^μ_15_ (**figure 3**).

These two sets of four variables were used as input predictors to build classification linear models based on the GDA implemented in the *STATISTICA* software [34]. The alignment-free classifiers based on Nandy-like structures provided classification accuracy in training and test of 84.87 and 84.95%, respectively. The AUC and F-score for the test set were of 0.919 and 0.687, respectively. In contrast, the TIs derived from the Mfold structures showed a better classification performance. Its accuracy level was notably higher in training (94.17%) and in the test subset (94.26%). The same was true for the AUC and F-score statistics that reach values of 0.983 and 0.960, respectively. These facts point out that the TIs calculated from the 2D topology predicted by folding thermodynamics rules are more effective classifiers than the TIs derived from artificial structures. However, the former takes much more computational and procedure cost than for the TIs obtained from the Cartesian graphical approach. The 2D Cartesian TIs have been useful for protein and RNA structure descriptors when higher structural levels are not available [46], [47], [48]. Thus, we evaluate non-linear methods on both data sets with the aim to improve the classification performance, especially for the pseudo-folding TIs. The Artificial Neural Networks (ANN), particularly the Multilayer Layer Perceptron (MLP) was selected as the most popular ANN architecture in use today [49].

#### 6.1 Artificial Neural Networks (ANN) in the prediction of the ITS2 class

The MLP was tested at different topologies using the four predictors already selected for each secondary structural approach as input variables. From the same training set used to develop the discriminant function, an independent data set (the selection set) was selected. This subset was chosen randomly taking out the 20% of the training set being not used in the back propagation algorithm. Thus, 12,168 cases were used for the training, 3,042 represented the selection subset and the 3,802 cases were evaluated in external validation to set the comparison.

The **Table 1** shows the different MLP topologies used to select the right complexity of the ANN in both datasets, the performance on training, selection and test progress were examined as well as its errors. The best models were the MLP profiles number 3 and 1 (highlighted in bold) for Nandy and Mfold datasets, respectively, which showed the best accuracy on training, selection and test sets, minimizing its respective errors. These ANN-models showed a higher accuracy level in classifying the training and test sets in respect to the linear models. The TIs calculated from the Mfold structures provided a better ANN performance on the data classification than when derived from the Nandy graphical approach. Although, ANN-based models showed an analogue behaviour in respect to the linear models (Mfold > Nandy); the classification performances of both structural approaches are more similar and higher when a non-linear function is applied (**Table 1**). This suggests that the identification of gene signatures tend to be better assessed with non-linear models and we further showed the utility of the artificial but informative folding of the biopolymeric sequences for gene/protein class identification [24], [50], [51].

**Table 1:** Testing different topologies for the MLP on the ITS2 classification using TIs from Nandy and Mfold DNA structures.

Nandy structure							
	Profile	Train Accuracy	Selection Accuracy	Test Accuracy	Train Error	Selection Error	Test Error
**1**	MLP 4:4-4-1:1	0.946	0.948	0.946	0.232	0.226	0.230
**2**	MLP 4:4-3-1-1:1	0.946	0.949	0.945	0.225	0.219	0.224
**3**	**MLP 4:4-2-2-1:1**	**0.959**	**0.958**	**0.956**	**0.178**	**0.180**	**0.187**
**4**	MLP 4:4-1-3-1:1	0.949	0.950	0.948	0.199	0.198	0.200
**5**	MLP 4:4-3-1:1	0.946	0.948	0.946	0.232	0.226	0.230
**6**	MLP 4:4-2-1-1:1	0.772	0.769	0.768	0.419	0.422	0.422
**7**	MLP 4:4-1-2-1:1	0.946	0.949	0.945	0.216	0.210	0.215
**8**	MLP 4:4-2-1:1	0.946	0.948	0.946	0.232	0.225	0.230
**9**	MLP 4:4-1-1:1	0.946	0.949	0.945	0.233	0.226	0.231

Mfold structure							
**1**	**MLP 4:4-4-1:1**	**0.976**	**0.975**	**0.973**	**0.140**	**0.138**	**0.145**
**2**	MLP 4:4-3-1-1:1	0.968	0.968	0.967	0.158	0.155	0.162
**3**	MLP 4:4-2-2-1:1	0.942	0.954	0.943	0.207	0.196	0.204
**4**	MLP 4:4-1-3-1:1	0.941	0.955	0.943	0.206	0.194	0.203
**5**	MLP 4:4-3-1:1	0.969	0.970	0.967	0.159	0.155	0.162
**6**	MLP 4:4-2-1-1:1	0.957	0.961	0.960	0.176	0.170	0.172
**7**	MLP 4:4-1-2-1:1	0.943	0.955	0.944	0.205	0.193	0.202
**8**	MLP 4:4-2-1:1	0.943	0.956	0.944	0.205	0.193	0.202
**9**	MLP 4:4-1-1:1	0.941	0.940	0.945	0.209	0.211	0.199

*Accuracy and error rates on training, selection and test sets.*

The classification results derived from our two best alignment-free approaches to classify ITS2 membership is showed in **Table 2** **and File S3**. The structural TIs based on the folding thermodynamics rules provide a more accurate description of the DNA/RNA structure, which is supported by the classification results (**Table 2**). The 2D topology of these molecules is affected by the primary information and by the possible hydrogen interactions between nucleotides forming the stems and loops; therefore a better functional classification performance is achieved. Although the Nandy-like representation is less accurate in the classification due to its artificial nature, it takes into account the sequence order information and the nucleotide composition, which are important features for the recognition at a genome scale of genes that do not encode a protein [52], [53]. Thus, the utility of this easy structural approach is reflected in the excellent discrimination achieved between these two distinct DNA/RNA functional classes with divergence among its members but sharing common structural features.

**Table 2:** Classification results derived from the ANN-models (MLP-3 and 1) for Nandy and Mfold structures respectively in training, selection and test series.

Nandy structure	Training			Selection		Test			
	ITS2		CG	ITS2	CG	ITS2		CG	
ITS2 class	**2434**		128	**575**	31	**770**		38	
Control Group (CG)	368		**9238**	87	**2349**	121		**2863**	
Total	2802		9366	662	2380	891		2911	
Sensitivity (Sv) (%)	86.86			86.85		86.42			
Specificity (Sp) (%)	98.63			98.70		98.35			
Accuracy (Acc) (%)	95.95			96.12		95.58			
AUC	0.984			0.985		0.980			
F-score						0.939			
**10-fold CV**	**Sv**	**Sp**	**Acc**			**Sv**	**Sp**	**Acc**	**AUC**
Average	84.79	98.85	95.64			84.59	98.87	95.59	0.978

Mfold structure	Training			Selection		Test			
	ITS2		CG	ITS2	CG	ITS2		CG	
ITS2 class	**2592**		102	**604**	19	**825**		35	
Control Group (CG)	210		**9264**	58	**2361**	66		**2876**	
Total	2802		9366	662	2380	891		2911	
Sensitivity(Sv) (%)	92.50			91.24		92.59			
Specificity (%)	98.91			99.20		98.79			
Accuracy (%)	97.57			97.53		97.31			
AUC	0.994			0.995		0.994			
F-score						0.960			
**10-fold CV**	**Sv**	**Sp**	**Acc**			**Sv**	**Sp**	**Acc**	**AUC**
Average	92.37	99.01	97.50			92.26	98.97	97.44	0.993

10-folds Cross Validation (CV) procedure on training and test sets.

*Numbers in bold highlight well-classified cases.*

We carried out a 10-fold cross validation to examine the classification performance of our alignment-free models. This validation procedure is easier to implement and provides reliable results in the validation of a predictive model at low computational cost [54]. Thus, the original data set was divided at random into 10 subsets containing the same number of cases. Of the 10 subsets, a single subset was retained as a prediction subsample for testing the model, and the remaining nine subsets were used as the training data. Since a selection subset is also needed to check the training algorithm, it was selected from the training set at random (10%). The cross-validation procedure is then repeated 10 folds or rounds using each of the 10 subsets for prediction exactly once, in such way ensures that all cases were predicted and used in training. Afterwards the average values for the accuracy, sensitivity, specificity for training and test sets, as well as the AUC were calculated to provide a single estimation from the 10 folds (**Table 2**).

**Figure 4:**
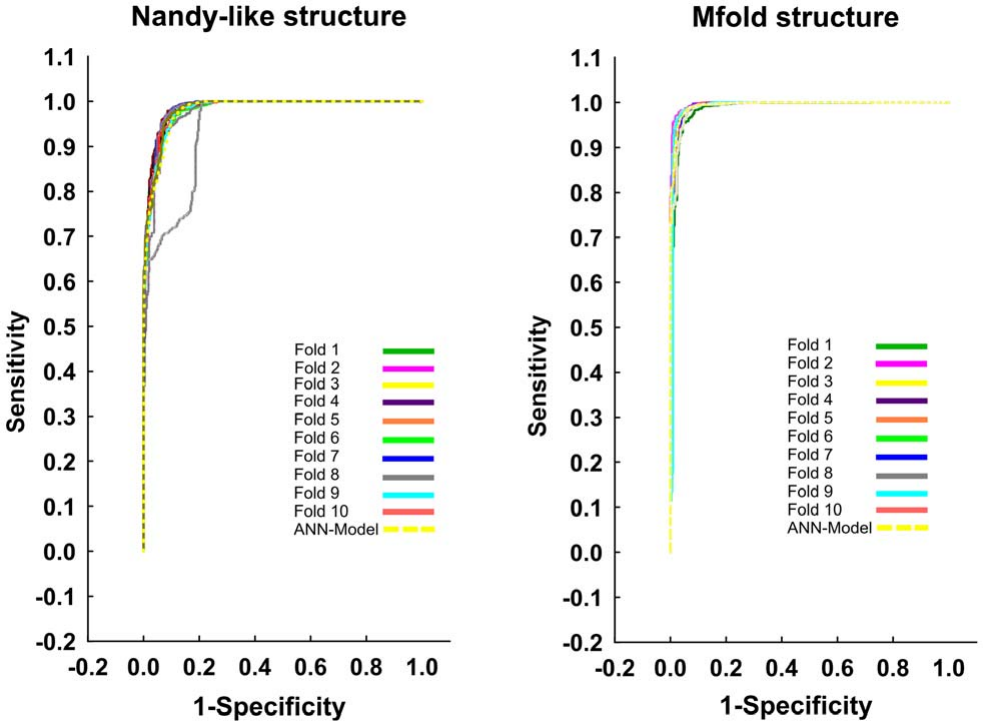
ROC-curves for the 10-fold cross validation procedure of both ANN-models (Nandy and Mfold structures) on the test set. The curve for the reported model in each case is represented by a yellow discontinuous line.

We plotted the ROC curve for each fold from the cross-validation procedure on the test set. In each fold or round, the curve presented an area higher than 0.5 (**figure 4**). According to the ROC curve theory random classifiers have an area of only 0.5. This result confirms that the present model is a significant classifier relatively to those working at random. In the plotting, the ROC curves for the ANN-models (MLP-1 and 3) on the test set were included to show visually its classification performance similarity with the 10-fold cross validation (**figure 4**). Thus, the similarity in the prediction performance between the 10-fold cross validation procedure and the reported ANN-models shows the robustness of our models. The validity of this type of procedures in structure-function relationship studies based on ANN-models has been demonstrated before [55], [56], [57].

We found an optimum cutoff for ITS2 gene classification using an “acceptance” threshold of 0.475 that provides a sensitivity of 0.929 and a specificity of 0.986 for our best predictive model (based on M-fold' TIs). Moreover, for the other alignment-free model that used Nandy-like's TIs, the “acceptance” classification threshold was 0.529 showing a sensitivity of 0.838 and a specificity of 0.988.

**Figure 5:**
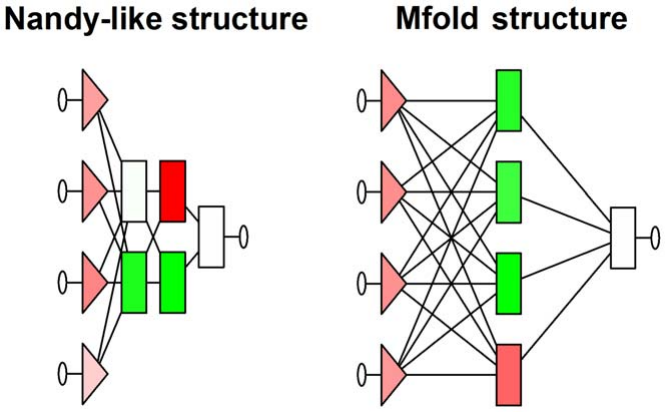
The architecture of the ANN-models (MLP-3 and MLP-1) for Nandy and Mfold structures, respectively. It represents four input variables, four neurons in two layers (Nandy) and four in one layer (Mfold) and only one output variable (from the left to the right).

Although ANN-based models are more complex than linear functions, the architecture of these networks is rather simple since they use just four predictors and one hidden layer made up of four neurons for the case of the TIs calculated from Mfold structures and two layers with the same amount of neurons for the Nandy structural approach (**figure 5**). Thus, the ANN-models based on the TI2BioP methodology are effective and simple tools to search an ITS2 sequences among the diversity of this DNA/RNA class in a wide variety of eukaryotic taxa.

### 7. Hidden Markov Models in the classification of the ITS2 class. A comparative study

Hidden Markov Models (HMM) has been widely used for classification purposes of DNA and protein sequences [58]. Their simplicity and high performance have made them the core of the popular database Pfam [4]. Profile HMMs generates predictive models in which classification performance can be easily evaluated in terms of accuracy, sensitivity and specificity. Nine profile HMMs from members of the ITS2 class were built up using three MSA algorithms (CLUSTALW, DIALIGN-TX and MAFFT) with different training sets. The classification measures for both the profile HMMs and the alignment-free models are shown in **Table 3**.

**Table 3:** Comparative analysis for the classification performance on the test set and *Petrakia sp.* ITS2 sequence using nine profile-HMMs built up with CLUSTALW, DALIGN-TX and MAFFT algorithms with different training sets.

ALIGNMENT BASED MODELS				
Training set (source and number of sequences)	Sequence Alignment (processing) Method	Optimal Classification Cutoff (E-value)	Sensitivity/Specificity (%)	Prediction on the ITS2 *Petrakia* sp.*
Representative fungi (80 sequences)	CLUSTALW	2.0	15.82/100	No significant hit
	DALIGN-TX	9.0	18.18/100	No significant hit
	MAFFT	5.0	20.20/100	0.02
Representative eukaryotes (134 sequences)	CLUSTALW	2.0	13.92/100	No significant hit
	DALIGN-TX	0.1	6.95/100	No significant hit
	MAFFT	2.0	3.59/100	No significant hit
Eukaryotes (2802 sequences)	CLUSTALW	8.0	12.69/100	No significant hit
	DALIGN-TX	0.8	35.58/100	No significant hit
	MAFFT	4.0	66.66/100	1.0

ALIGNMENT-FREE MODELS				
Training set (source and number of sequences)	2D Structural Approach	Optimal Classification Cutoff (Accept/Reject)	Sensitivity/Specificity (%)	Prediction on the ITS2 *Petrakia* sp.
Eukaryotes (12168 sequences)	**Nandy structure**	Accept > 0.529	83.80/98.80	0.990
	**Mfold structure**	Accept > 0.475	92.90/98.60	0.996

The classification results of our alignment-free models (Mfold and Nandy) when using an optimal cutoff are also provided.

*Classification performance at optimal cutoff in every case (E-value).

As shown in **Table 3**, all the profile HMMs obtained for the ITS2 classification provide a lower performance in respect to the alignment-free approaches. Nevertheless, we obtained generally some improvements in the sensitivity on the ITS2 classification when the E-value cutoff was increased (**File S6**) and when the profile HMMs based on improved MSA algorithms was applied. The use of a wider training set comprising 2802 ITS2 sequences also improved the classification performance for the profile HMMs based on DIALIGN-TX and MAFFT algorithms since this dataset better captures the vast diversity of the ITS2 class. However, the ITS2 query sequence from *Petrakia* sp. was identified with a higher significance level when a fungi-specific dataset aligned with MAFFT was considered for building the models (**Table 3**).

We provide information about the MSA handled with CLUSTALW, DIALIGN-TX and MAFFT (**File S4**) and the ITS2 profile HMMs generated with the aforementioned MSA algorithms on the three training sets described in section 2.3 (**File S5**).

We explain the low performance of the profile HMMs on the poorly informative multiple alignments used for its creation. Neither the use of a specific nor of an extended training set aligned with an improved MSA (e.g. MAFFT) assures a good classification; the maximum sensitivity obtained on the test set was only 66.66% (**Table 3**). This result is in line with the one previously obtained by developers of the ITS2 database [10], which reported the use of more conserved 5.8S and 28S rRNAs adjacent to the ITS2 in order to obtain an useful profile HMM. All together, these results reinforce the usability of our alignment-free models that additionally require less sequence information compared to classical alignment-based approaches.

As a practical validation, a novel ITS2 genomic sequence was isolated from a fungal isolate as a part of its taxonomic characterization. This ITS2 sequence was used to evaluate the ability of the ANN-models and the profile HMMs to identify a novel member of this gene class and also its use into the traditional and alignment-free phylogenetic assessment.

### 8. Experimental results. Annotation of a novel ITS2 member using several predictive models

We selected the fungal genus *Petrakia* that lives inside plants of the *genus Acer*, which can be a latent pathogen agent of these plants and a potentially producer of bioactive compounds [59]. Members of the *Petrakia* genus are placed inside the Ascomycota phylum despite the absence of a defined ascus (a microscopic sexual structure in which nonmotile spores, called ascospores, are formed). These fungi that produce conidia (mitospores) instead of ascospores were previously described as mitosporic Ascomycota [53]. However, its taxonomy identification has been a problem at the species level. Thus, a polyphasic approach involving mycological culture with molecular detection [60] to determine the presence of fungi in plants is needed.

**Figure 6:**
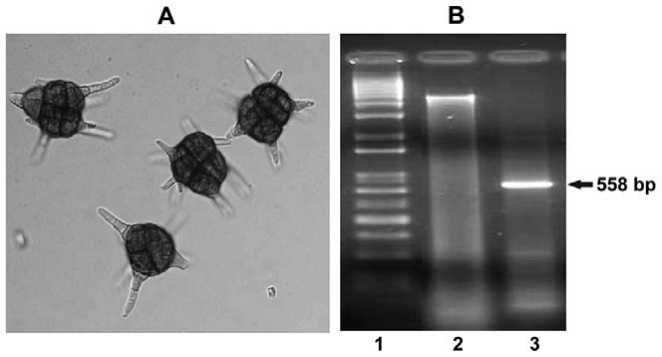
Conidia of Petrakia sp. from 7 days culture on Malt Extract Agar (×400) (A). Isolation of a novel ITS2 genomic sequence from *Petrakia* sp. (1) 1 Kb ladder (Gibco BLR), (2) Genomic DNA from the *Petrakia* isolate, (3) PCR reaction with the ITS5 and ITS4 primers (B).

Our fungal isolate showed all morphological characteristics of a mitosporic Ascomycota/ genus *Petrakia* such as: aerial mycelium, cover entire plate of Malt Extract Agar medium, conidiophores forming dark sporodochium, conidia pigmented, many-celled, muriform, with several cylindrical projections [61] (**figure 6A**). However, the species could not be unequivocally determined and therefore an attempt to perform a low level-phylogenetic analysis supported on the ITS2 biomarker was required to complement the fungus detection.

We isolated a genomic DNA fragment of 558 bp comprising the entire (ITS1, 5.8S rDNA, and ITS2) region with shorts ends at 5′and 3′positions corresponding to the 18S and 28S rDNA conserved genes, respectively (**figure 6B**). The PCR product was sequenced and registered at the GenBank Database (accession number FJ892749). The ITS2 region was delineated by alignment methods [62] using the conserved 5.8S and 28S rDNA flanking fragments. Then, the ITS2 region was selected to evaluate the predictability of our alignment-free models based on the TI2BioP methodology and also by predictive alignment procedures.

We selected the ANN-based models for the ITS2 classification since they show the highest classification rate for both structural approaches. Both alignment-free models allowed a successfully prediction of the *Petrakia* ITS2 sequence with a confidence level of 0.996 and 0.990 for the Mfold and Nandy-like structures, respectively (**Table 3**). Despite the high divergence among the ITS2 sequences, the models were able to identify a new fungal ITS2 sequence from a dataset made up of divergent UTR sequences with similar structural features but functional different. We also demonstrated that Nandy-like structures provide patterns that are useful for gene class discrimination. These 2D artificial maps for DNA/RNA provides information about the connectivity of the nucleotides, but also accounts for the content of purines (GA) and pyrimidine (CT) in the rDNA molecules, which can be observed in the tendency of occupying certain quadrant in the Cartesian system (**figure 1**). The variations in the content of nucleotides have been also used in the genomic recognition of non-protein-coding RNAs [52].

By contrast, profile HMMs generated with different MSA algorithms and different training sets showed in general a poor classification performance on the ITS2 sequence of *Petrakia* sp. Only the profile HMMs based on MAFFT classified it correctly (**Table 3**). Despite that the alignment-free methods and the profile HMMs based on MAFFT recognized our query ITS2 sequence with significance, a BLASTn search (*E*-value cutoff = 10e^−10^) against the NCBI database was carried out to support the annotation of the newly isolated sequence by looking for hits belonging or related to the *Petrakia* genus. We retrieved the second best hit (HQ433006) from an uncultured fungus from the Ascomycota phylum. The score (172) and sequence similarity (89%) between our query and this hit were significant (*E*-value = 4e-^40^). However, the BLAST search did not find any hit from the *Petrakia* genus except our own submission (first hit). This confirms that *Petrakia* genus is not well-represented at NCBI and has not been deeply studied yet either taxonomically or as a source of novel secondary metabolites.

### 9. A comparative phylogenetic analysis

The lack of other ITS2 sequences from different species of the genus *Petrakia* (with the exception of our sequence submission at the GenBank) precluded performing a phylogenetic analysis at the species level (low-level analysis). We classified our fungal isolate as a mitosporic Ascomycota/*Petrakia sp.* according to its mycological culture features, as there is not a report with a detailed taxonomy about this genus namely in the NCBI dedicated ‘Taxonomy’ database (http://www.ncbi.nlm.nih.gov/tanonomy). Furthermore, there is no specification about its subphylum and class [63]. These fungal species was initially placed into a separate artificial phylum “the Deuteromycota” along with asexual species from other fungal taxa but currently asexual ascomycetes are identified and classified based on morphological or physiological similarities to ascus-bearing taxa, as well as based on phylogenetic analyses of DNA sequences [64]. So, a higher-level phylogenetic study involving Ascomycota members haring ITS2 sequence similarities with *Petrakia* may complement its taxonomy relatively to the ascus-bearing taxa. First, we assumed that our fungal isolate belonged to the *Pezizomycotina* subphylum, the largest within Ascomycota phylum. Our inference agree with a recent classification found in the “The dictionary of the Fungi” [65].

**Figure 7:**
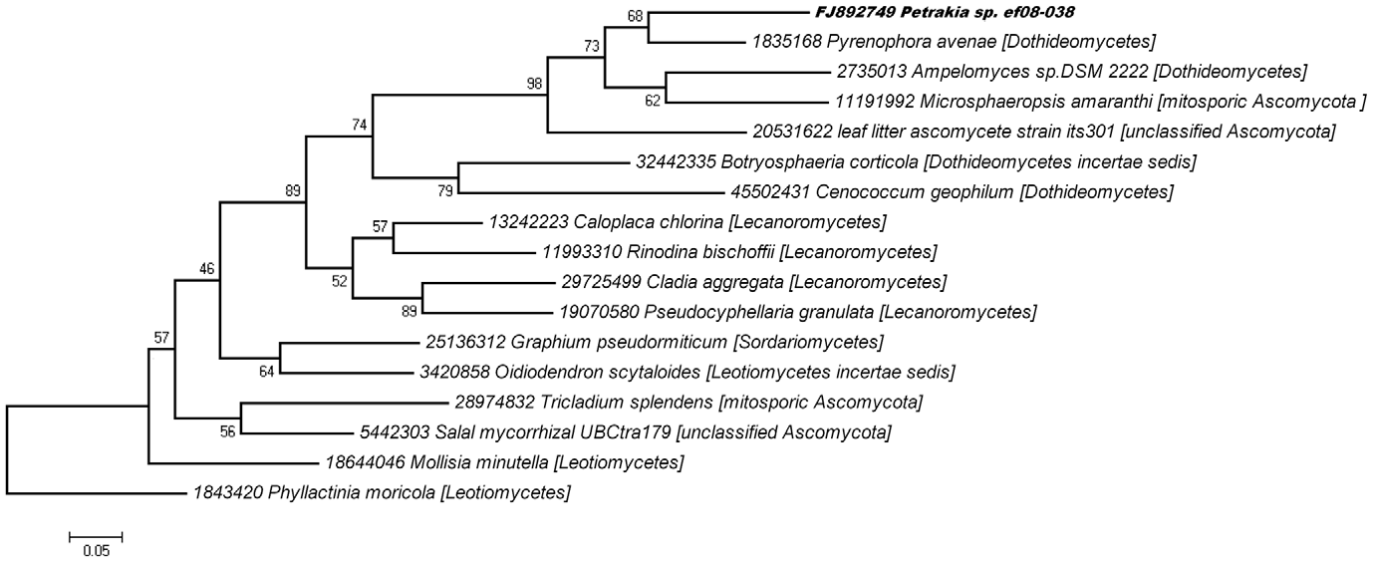
Neighbor-joining tree based on the ITS2 sequences using the substitution Kimura 2-parameter (K2P).

Two different types of distance trees were built: (1) a traditional one based on multiple alignments of ITS2 sequences and (2) another irrespective of sequence similarity supported by the TI2BioP methodology. Both phylogenetic analyses, the traditional and the alignment-free clustering, showed that the *Petrakia* isolate is similar to the Dothideomycetes class members (**figure 7** **and** **8**). Dothideomycetes is the largest and most diverse class of ascomycete fungi. They are often found as pathogens, endophytes or epiphytes of living plants sharing some morphological features described above for the *Petrakia* genus [66]. In addition, *Petrakia sp.* was placed by the two different computational taxonomic approaches near to the mitosporic Ascomycota *Ampelomyces sp.DSM 2222* supporting the mycological characterization of the query fungus. *Ampelomyces sp.DSM 2222* is taxonomically placed among the Dothideomycetes class and inside the mitosporic Leptosphaeriaceae family producing conidia as *Petrakia sp.* We only show the NJ-tree based on the K2P substitution model to illustrate the tree topology and the BS values for each node that support our phylogenetic inferences (**figure 7**). Similar tree topologies and BS support were obtained with other evolutionary distance matrices and the ME method (see section 2.4) (**figure S2**).

**Figure 8:**
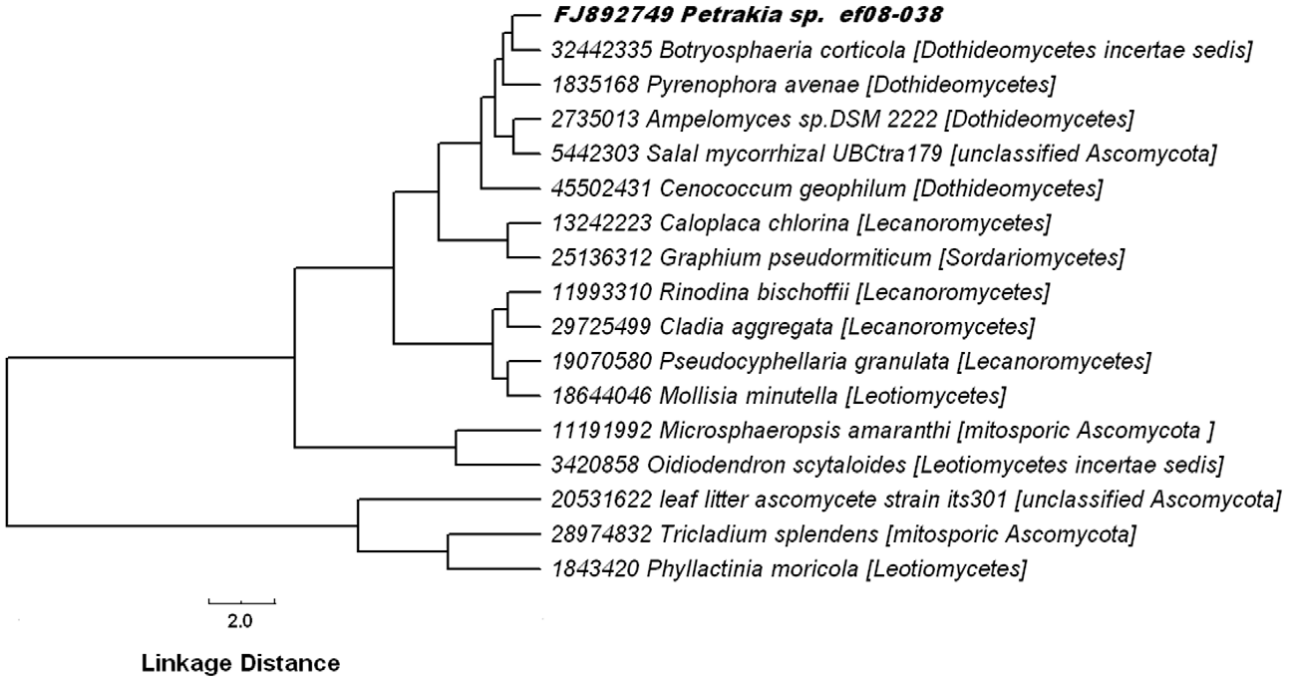
Neighbor-joining tree clustering based on the Euclidean distance calculated from the TIs values.

Furthermore, we evaluate the stability of our results on the NJ-tree clustering: (i) by measuring the influence of several alignment-free distances (City-block, Chebychev, and Power distance) in addition to the Euclidean distance, (ii) by assessing other clustering methods (Single linkage, Uweighted pair-group average and the Ward's method) and (iii) by calculating the cophenetic correlation coefficient for the clustering depicted in the **figure 8**. The topologies of the alignment-free trees based on different distance metrics are quite similar as well as the positions of the taxa in respect of our query fungus along the four trees (**figure S3**). Similar outcomes were obtained when different clustering methods were computed using the Euclidean distance to plot the trees (**figure S4**). These two facts support the consistency of our original alignment-free clustering despite the difficulty to perform a statistical significance testing, as unlike many other statistical procedures, cluster analysis methods are mostly used when we do not have any *a priori* hypotheses. One way to measure the validity of the cluster information generated by the linkage function is to compare it with the original proximity data generated by the pairwise distance (Euclidean) function. If the clustering is valid, the linking of objects in the cluster tree should have a strong correlation with the distances between objects in the distance vector. The cophenet function compares these two sets of values and computes their correlation, returning a value called the cophenetic correlation coefficient (ccc) [18]. We retrieve a ccc value for the furthest-neighbor clustering of 0.87 showing an strong correlation (the closer the value of the ccc is to 1, the better the clustering solution). The cophenet function was used to evaluate the clustering method using the other distance metrics mentioned above. The ccc values for the City-block, Chebychev, and Power distances were 0.84, 0.82 and 0.80, respectively, showing consistency in the clustering solution.

The tree topologies obtained for both approaches are somewhat similar as well as the sub-topologies within the Ascomycota classes, specially the Petrakia's location among the Dothideomycetes. Moreover, *Phyllactinia moricola* (outgroup) is placed far from the rest of the members (inner group). Therefore, the NJ clustering based on the Euclidean distance matrix computed from our alignment-free indices largely agrees with the traditional NJ distance tree, which have a phylogenetic meaning since is based on evolutionary distances.

These findings support the importance of including ITS2 structural information when assessing the phylogenetic relationships at higher levels in eukaryote evolutionary comparisons. Although the Euclidean distance is simply a sort of geometric distance in a multidimensional space with no phylogenetic meaning, it led to an effective hierarchical biological clustering with an evolutionary approach because it was derived from the TIs containing both sequence and structural information.

### Conclusions

Topological indices containing information about ITS2 sequences and structures are effective to produce ANN-models with a high prediction power despite the sequence diversity of this class. The use of artificial but informative DNA/RNA secondary structures is a less-costly alternative for the ITS2 classification when higher structural levels are not available or the correct structure is only rarely found by standard RNA folding algorithms. TI2BioP provided simplicity and reliability to ANN-models to search a novel ITS2 member, performing even better than the profile HMMs built up with optimized MSA algorithms for low overall sequence similarity. In addition, our alignment-free approach is effective to construct hierarchical distance-trees containing relevant biological information with an evolutionary significance.

## Supporting Information

File S1 Exploring ITS2 and UTRs sequence diversity by Needleman-Wunsch and Smith-Waterman procedures.
(DOC)

File S2 IDs, training and prediction series, values of the TIs predictors for the ITS2 and UTR sequences.
(XLS)

File S3 Classification results derived from ANN-models on the training, selection and test set for the two structural approaches.
(XLS)

File S4 MSA performed by several algorithms (CLUSTALW, DIALIGN-TX and MAFFT) using three different training sets (File S4.1–4.9).
(RAR)

File S5 ITS2 profile HMMs generated with the MSA showed in File S4 (File S4.1–4.9).
(RAR)

File S6 ROC analysis for each profile HMM at 20 different E-values (0.1–10).
(XLS)

Figure S1 Pair wise comparison (all *vs* all) for the ITS2 and UTRs sequences evaluated in this study using the Needleman-Wunsch (NW) (in light gray) and Smith-Waterman (SW) (in dark gray) alignment algorithms.
(TIF)

Figure S2 Neighbor-joining trees based on JC (in black) and MCL (in red) substitution models and ME trees based on the JC (in green) and K2P (in blue) evolutionary distances.
(TIF)

Figure S3 Neighbour-joining trees built with different alignment-free distance metrics: Euclidean (in black), City-block (in blue), Chebychev (in red) and Power (in green) distances. Each taxa is labeled for a number as follow: (1) FJ892749 *Petrakia sp. ef08-038*, (2) 1835168 *Pyrenophora avenae* [Dothideomycetes], (3) 2735013 *Ampelomyces sp.DSM 2222* [Dothideomycetes], (4) 11191992 *Microsphaeropsis amaranthi* [mitosporic Ascomycota], (5) 20531622 *leaf litter ascomycete strain its301* [unclassified Ascomycota], (6) 32442335 *Botryosphaeria corticola* [Dothideomycetes incertae sedis], (7) 45502431 *Cenococcum geophilum* [Dothideomycetes], (8) 13242223 *Caloplaca chlorina* [Lecanoromycetes], (9) 11993310 *Rinodina bischoffii* [Lecanoromycetes], (10) 29725499 *Cladia aggregata* [Lecanoromycetes], (11) 19070580 *Pseudocyphellaria granulata* [Lecanoromycetes], (12) 25136312 *Graphium pseudormiticum* [Sordariomycetes], (13) 3420858 *Oidiodendron scytaloides* [Leotiomycetes incertae sedis], (14) 28974832 *Tricladium splendens* [mitosporic Ascomycota], (15) 5442303 *Salal mycorrhizal UBCtra179* [unclassified Ascomycota], (16) 18644046 *Mollisia minutella* [Leotiomycetes], (17) 1843420 *Phyllactinia moricola* [Leotiomycetes].
(TIF)

Figure S4 Joining-tree clustering using different methods for the linkage of the Euclidean distance: Complete linkage (in black), single linkage (in blue), unweighted pair-group average (in red) and the Ward's method (in green). Taxa are labeled by numbers as in the figure S3.
(TIF)
