# Supplementary material for: An Alignment-Free Approach for Eukaryotic ITS2 Annotation and Phylogenetic Inference
Source: PLoS One. 2011 Oct 26;6(10):e26638. doi: 10.1371/journal.pone.0026638 (PMC3202569; doi:10.1371/journal.pone.0026638)
Supplement: File S1 — Exploring ITS2 and UTRs sequence diversity by Needleman-Wunsch and Smith-Waterman procedures. (DOC) [file pone.0026638.s001.doc]

***Exploring ITS2 and UTRs sequence diversity***

In order to explore the diversity of both groups of sequences, the Needleman-Wunsch and Smith-Waterman dynamic alignment algorithms were assessed independently on the 4 356 ITS2 and 14 657 UTRs sequences (all *vs* all). We included among the ITS2 dataset, a new ITS2 genomic sequence from our fungal isolate of the *Petrakia* genus. The aligned pairs were clustered using the values of the similarity matrix and the number of sequence pairs was plotted *vs* similarity percentage into a histogram for the two alignment algorithms using the Mathlab software **(Figure S1).** For the ITS2 class, both algorithms agree that the major amount of sequences pairs shared similarities between 40 and 50% decreasing at lower and higher values of similarity percentages. Since the SW procedure finds the optimal local alignment between two sequences, the histogram peak is shifted to the right (highest values of similarity percentages); even finding sequences pairs sharing similarities percent below 25% is odd. However the NW algorithm showed a symmetric histogram with pairs of sequences with similarities values from near to zero to values higher than 95.0%. The UTR dataset had a similar behavior when these two alignment procedures were applied on it. However, it showed a lowest similarity (30-40%) percentage in respect to the ITS2 class for the major number of sequences pairs. The dissimilarity among the UTRs pairs is higher than the presented for the ITS2 sequences since the UTR dataset involved 5´and 3´ends of different fungal mRNAs. We can conclude that the two datasets involved in the study show a high sequence diversity among its members, which was demonstrated comparatively by two dynamic alignment algorithms.

**Figure S1 comes about here**

1. Needleman, S.B. and C.D. Wunsch, *A general method applicable to the search for similarities in the amino acid sequence of two proteins.* J Mol Biol, 1970. **48**(3): p. 443-53.

2. Smith, T.F. and M.S. Waterman, *Identification of common molecular subsequences.* J Mol Biol, 1981. **147**(1): p. 195-7.

3. Gilat, A., ed. *MATLAB: An Introduction with Applications*. 2nd ed. 2004, John Wiley & Sons.
