# Supplementary figures and images for: An Alignment-Free Approach for Eukaryotic ITS2 Annotation and Phylogenetic Inference
Source: PLoS One. 2011 Oct 26;6(10):e26638. doi: 10.1371/journal.pone.0026638 (PMC3202569; doi:10.1371/journal.pone.0026638)

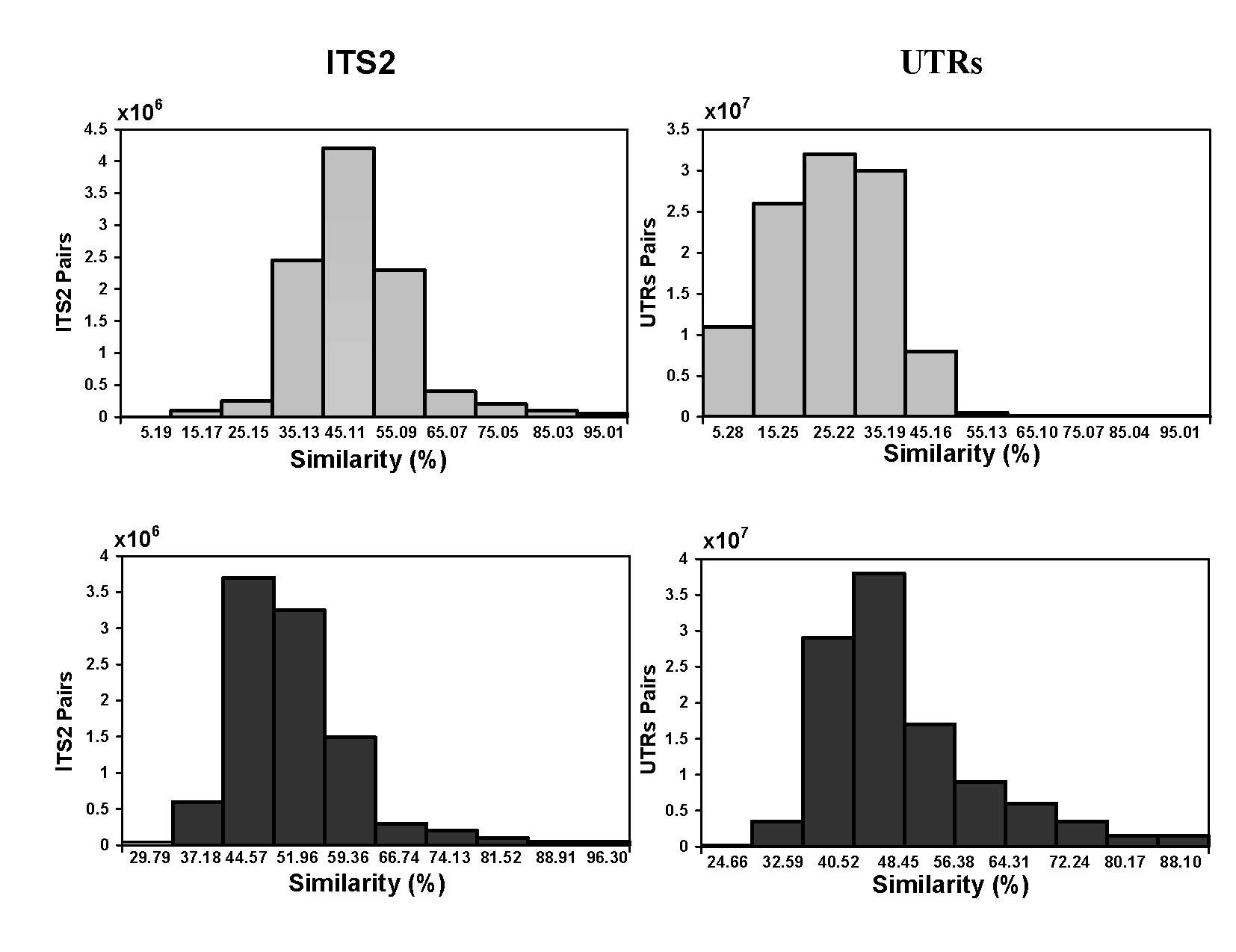

Supplement: Figure S1 — Pair wise comparison (all vs all) for the ITS2 and UTRs sequences evaluated in this study using the Needleman-Wunsch (NW) (in light gray) and Smith-Waterman (SW) (in dark gray) alignment algorithms. (TIF) [file pone.0026638.s007.tif]

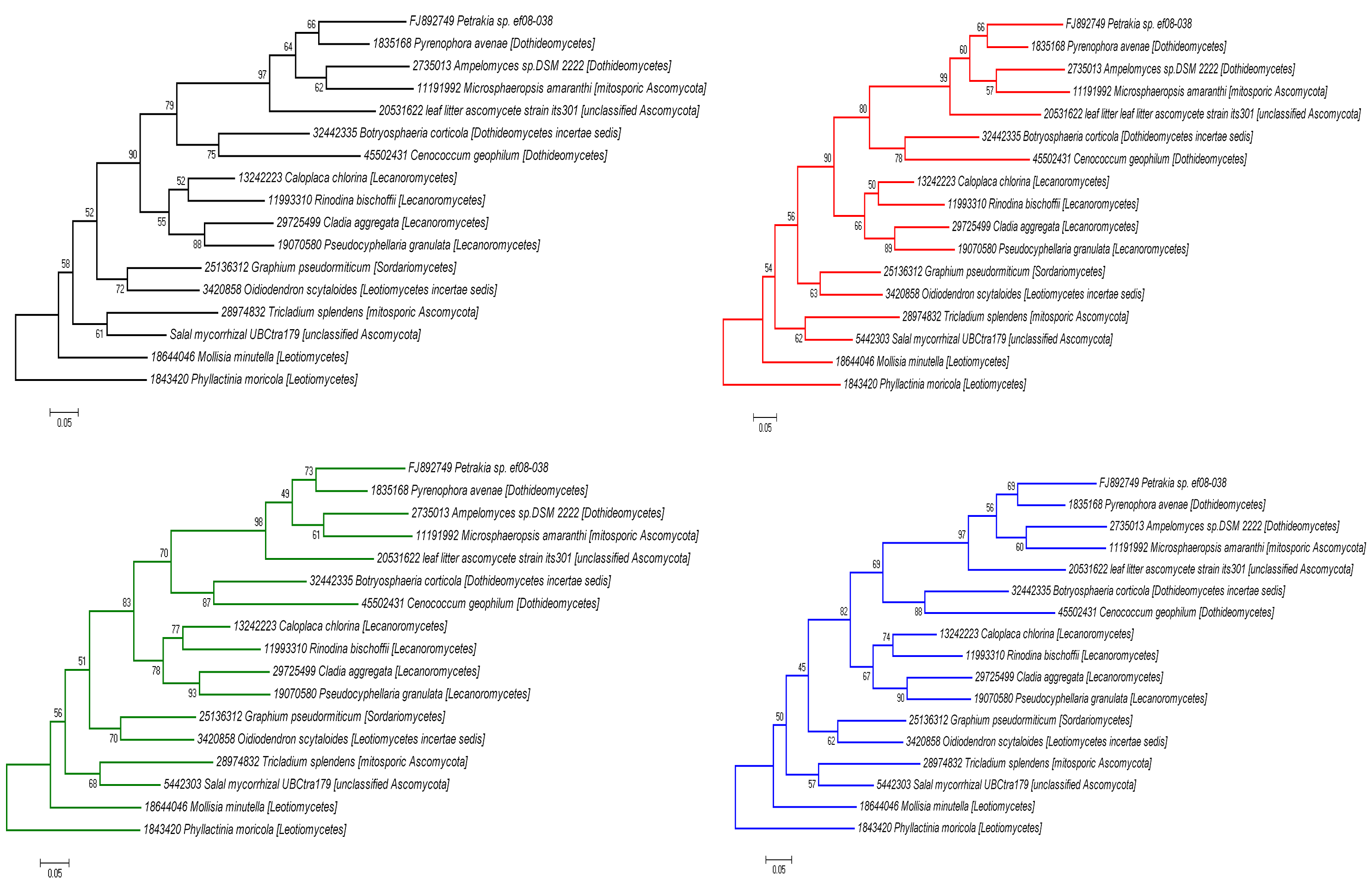

Supplement: Figure S2 — Neighbor-joining trees based on JC (in black) and MCL (in red) substitution models and ME trees based on the JC (in green) and K2P (in blue) evolutionary distances. (TIF) [file pone.0026638.s008.tif]

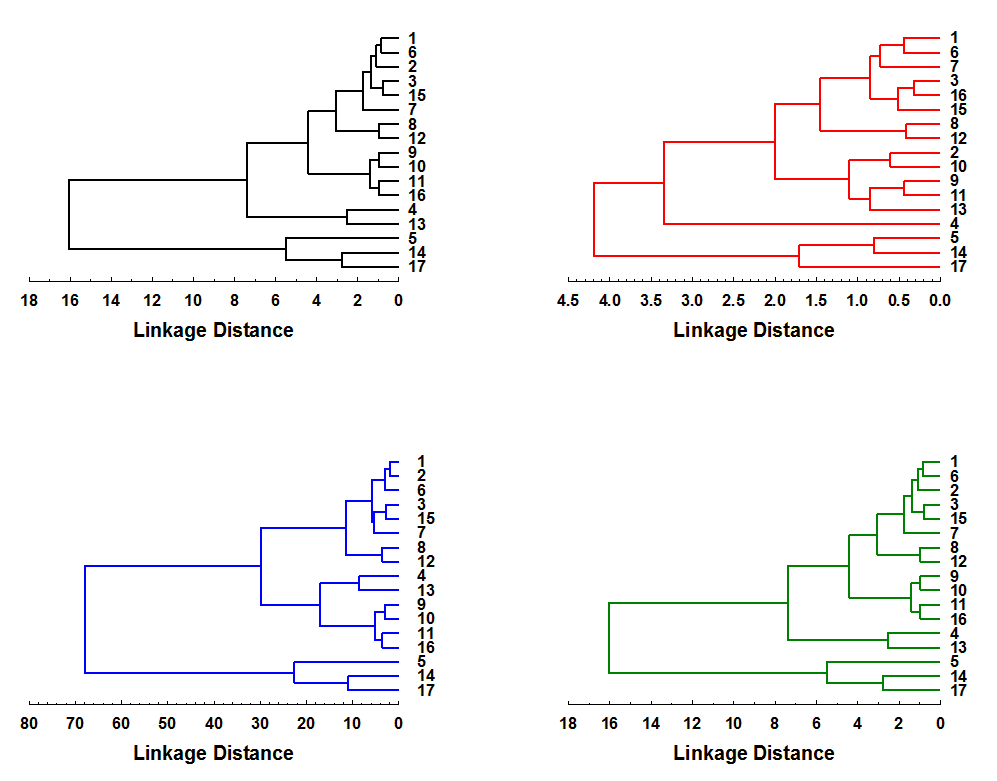

Supplement: Figure S3 — Neighbour-joining trees built with different alignment-free distance metrics: Euclidean (in black), City-block (in blue), Chebychev (in red) and Power (in green) distances. Each taxa is labeled for a number as follow: (1) FJ892749 Petrakia sp. ef08-038, (2) 1835168 Pyrenophora avenae [Dothideomycetes], (3) 2735013 Ampelomyces sp.DSM 2222 [Dothideomycetes], (4) 11191992 Microsphaeropsis amaranthi [mitosporic Ascomycota], (5) 20531622 leaf litter ascomycete strain its301 [unclassified Ascomycota], (6) 32442335 Botryosphaeria corticola [Dothideomycetes incertae sedis], (7) 45502431 Cenococcum geophilum [Dothideomycetes], (8) 13242223 Caloplaca chlorina [Lecanoromycetes], (9) 11993310 Rinodina bischoffii [Lecanoromycetes], (10) 29725499 Cladia aggregata [Lecanoromycetes], (11) 19070580 Pseudocyphellaria granulata [Lecanoromycetes], (12) 25136312 Graphium pseudormiticum [Sordariomycetes], (13) 3420858 Oidiodendron scytaloides [Leotiomycetes incertae sedis], (14) 28974832 Tricladium splendens [mitosporic Ascomycota], (15) 5442303 Salal mycorrhizal UBCtra179 [unclassified Ascomycota], (16) 18644046 Mollisia minutella [Leotiomycetes], (17) 1843420 Phyllactinia moricola [Leotiomycetes]. (TIF) [file pone.0026638.s009.tif]

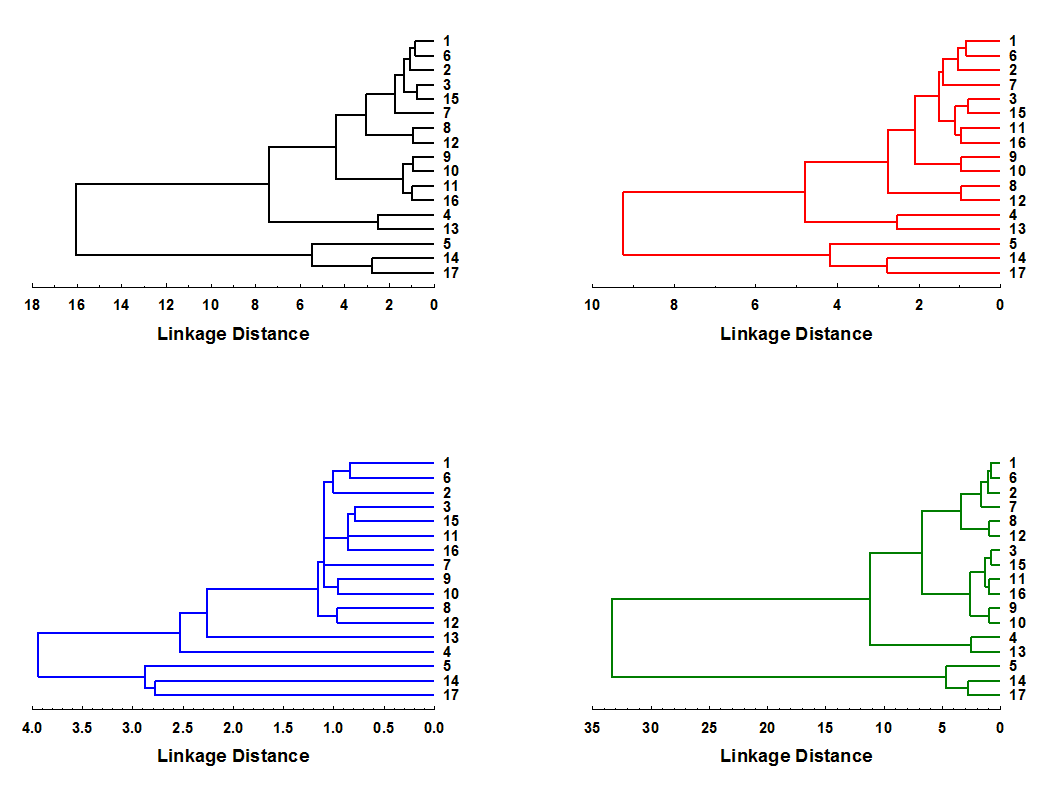

Supplement: Figure S4 — Joining-tree clustering using different methods for the linkage of the Euclidean distance: Complete linkage (in black), single linkage (in blue), unweighted pair-group average (in red) and the Ward's method (in green). Taxa are labeled by numbers as in the figure S3. (TIF) [file pone.0026638.s010.tif]
